# Activin receptor-like kinase 3: a critical modulator of development and function of mineralized tissues

**DOI:** 10.3389/fcell.2023.1209817

**Published:** 2023-06-30

**Authors:** Xianchun Ruan, Zhaowei Zhang, Munire Aili, Xiang Luo, Qiang Wei, Demao Zhang, Mingru Bai

**Affiliations:** ^1^ State Key Laboratory of Oral Diseases, West China Hospital of Stomatology, Sichuan University, Chengdu, China; ^2^ National Clinical Research Center for Oral Diseases, West China Hospital of Stomatology, Sichuan University, Chengdu, China; ^3^ West China School of Basic Medical Sciences and Forensic Medicine, Sichuan University, Chengdu, China; ^4^ Department of Cariology and Endodontics, West China Hospital of Stomatology, Sichuan University, Chengdu, China

**Keywords:** activin receptor-like kinase 3, bone morphogenetic protein, mineralized tissue, tooth, bone

## Abstract

Mineralized tissues, such as teeth and bones, pose significant challenges for repair due to their hardness, low permeability, and limited blood flow compared to soft tissues. Bone morphogenetic proteins (BMPs) have been identified as playing a crucial role in mineralized tissue formation and repair. However, the application of large amounts of exogenous BMPs may cause side effects such as inflammation. Therefore, it is necessary to identify a more precise molecular target downstream of the ligands. Activin receptor-like kinase 3 (ALK3), a key transmembrane receptor, serves as a vital gateway for the transmission of BMP signals, triggering cellular responses. Recent research has yielded new insights into the regulatory roles of ALK3 in mineralized tissues. Experimental knockout or mutation of ALK3 has been shown to result in skeletal dysmorphisms and failure of tooth formation, eruption, and orthodontic tooth movement. This review summarizes the roles of ALK3 in mineralized tissue regulation and elucidates how ALK3-mediated signaling influences the physiology and pathology of teeth and bones. Additionally, this review provides a reference for recommended basic research and potential future treatment strategies for the repair and regeneration of mineralized tissues.

## 1 Introduction

Mineralized hard tissue is characterized by its rigidity, low permeability, and poor blood flow, which makes it more challenging to repair than soft tissue (Shi et al., 2020). Despite the continuous remodeling of bones, the optimal treatment for critical-sized bone defects is still unclear, and the long-term outcomes of current treatments are limited by high rates of complications and reoperations (Nauth et al., 2018). Moreover, the regenerative potential of teeth is low or virtually absent since the stem cell populations capable of regeneration are lost at an early stage. Tooth defects and loss, which could result from tooth decay, congenital malformations, trauma, periodontal diseases, or age-related changes, are typically repaired by artificial materials lacking many important biological characteristics that natural teeth possess (Balic, 2018).

In the past decades, research on the molecular mechanisms underlying the development and homeostasis of mineralized tissue (i.e., tooth and bone) has progressed rapidly. Despite being independent tissues, tooth and bone share similar anatomical structures and physiological functions, with both consisting of a highly calcified outer structure and soft inner tissue and the inorganic component consisting of biological apatite (Abou Neel et al., 2016). Notably, the inner dentin-pulp layer of the tooth has mineralization characteristics similar to those of bone (Thrivikraman et al., 2019). Some researches suggest that there are common molecular pathways for the development of teeth and bones, such as Wnt/β-catenin (Duan and Bonewald, 2016), transforming growth factor β (Corps et al., 2021) and bone morphogenetic protein (BMP) signaling (Yuan et al., 2015; Wang et al., 2017). With the Col1a1-Cre in mice, activation of β-catenin led to increased ossification in long bone and vertebrae, delayed tooth eruption, and aberrant maxillofacial formation (Glass et al., 2005). The conditional knockout (cKO) of transforming growth factor β receptor II in mice using the Osterix (Osx)-Cre/loxP model resulted in defects in bones, reduced mineral density of the root dentin and shorter roots (Wang et al., 2013; Peters et al., 2017; Corps et al., 2021). Additionally, the deletion of BMP1 in mice led to abnormal odontoblast differentiation, short root dentin (Wang et al., 2017) and osteogenesis imperfecta (Muir et al., 2014). Due to the difficulty of bone and tooth regeneration, tissue engineering techniques are considered. Some implantable bioactive materials and growth factors are used to modulate the signaling pathways of the embedded cells and thus induce cell proliferation and differentiation to promote repair and regeneration of mineralized tissues.

For example, BMP2 as a growth factor is widely used in both dentin regeneration (Bakopoulou et al., 2016; Wang et al., 2016; Machla et al., 2023) and bone regeneration (Tan et al., 2019; Chen et al., 2021; Tateiwa et al., 2021). However, the specific signaling mechanisms underlying mineralized tissue repair are still unclear, and few reported signaling pathways have been used as molecular targets for clinical treatment. Therefore, it is crucial to find a new molecular target for promoting bone and tooth repair and regeneration.

BMPs are a highly conserved class of functional proteins involved in homeostasis of mineralized tissue, including bone formation (Sampath et al., 1993; Wu et al., 2016), bone repair (Salazar et al., 2016) and tooth development (Liu et al., 2022). About 15 BMP superfamily ligands have been identified. Previous studies have mainly focused on the roles of BMP2, BMP4, and BMP7 in bone formation and repair, but a large number of exogenous BMPs tended to accumulate clumps and caused inflammation and swelling, and conventional long-term use of BMP2 increased osteoclastic activity (Vukicevic et al., 2014; Gillman and Jayasuriya, 2021). Therefore, it is necessary to identify a more precise molecular target for hard tissue repair, such as BMP receptors downstream of BMPs. Different ligands can determine different cell fates and regulate the growth and development of different tissues by combining with different BMP receptors (Komorowski, 2022). The BMP receptor complex is composed of type I and type II serine-threonine kinase receptors. Four distinct BMP type I receptors (BMPRIs) and three BMP type II receptors (BMPRIIs), also termed activin receptor-like kinases (ALKs), have been characterized. Four type I receptors are found in mammalian genomes, namely, ALK1 (also termed ACVRL1), ALK2 (also termed ACVR1), ALK3 (also termed BMPRIa), and ALK6 (also termed BMPRIb); the three type II receptors are BMPR2, ActRIIA (also termed ACVR2a) and ActRIIB (also termed ACVR2b) (Gomez-Puerto et al., 2019). Type I receptors play a primary role in determining the specificity of intracellular signals. It is shown that ALK3 can selectively bind to BMP-2, -4, -5, -6, -7, and -8, and less efficiently to BMP-5, -9 and -10 (Heldin and Moustakas, 2016). However, ALK-3 cannot bind to BMP-1 because BMP-1 is an enzyme that participates in the degradation of extracellular matrix and protein modification processes (Kessler et al., 1996). In addition, although BMP-6 can bind ALK3; BMP2 and BMP-4 interact most strongly with ALK3. (Sanchez-Duffhues et al., 2020). BMP2 can further activate intracellular Smad1, Smad5 and Smad8 (Aoki et al., 2001). It is well known that FDA-approved BMP2 has started to be used in tissue engineering for bone repair and regeneration due to its osteo-inductive effects. Consequently, the high-affinity receptor ALK3 downstream of BMP2 deserves further attention. ALK3, a type I BMP receptor, is crucial for the growth and development of mineralized tissues. There have been many studies have shown that mice with ALK3 deficiency or inhibition exhibited impaired tooth formation (Kim et al., 2014), hypoplasia of the mandible (Li et al., 2011), and altered biomechanical properties of long bones (Zhang et al., 2016). Therefore, this review provides a retrospective summary and analysis of new ALK3-related findings in mineralized tissues. We aim to clarify the importance of ALK3 in tooth and bone regulation and provide recommendations for future basic research and potential biological target for tooth and bone repair and regeneration.

## 2 ALK3 and BMP signaling

**FIGURE 1:**
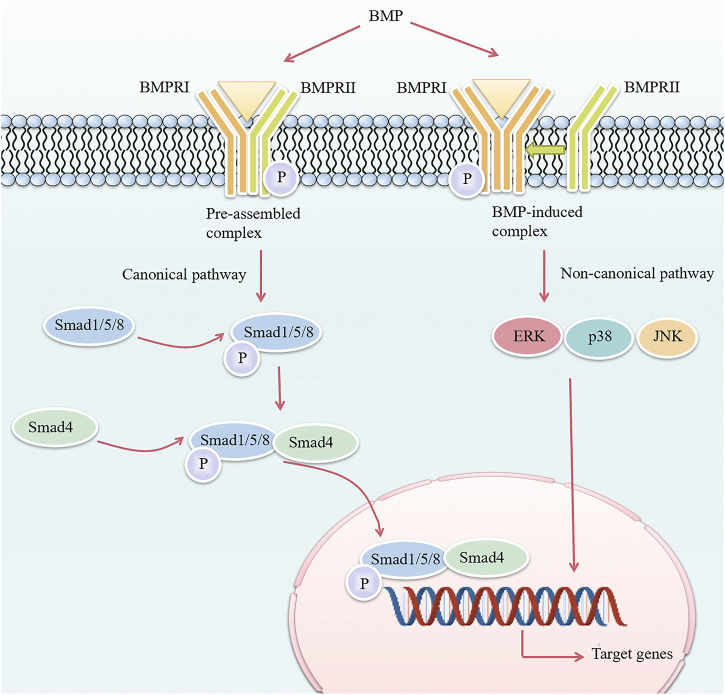
Overview of the BMP signaling pathway. The difference in the hetero-oligomerization pattern of cell surface receptors determines the activation of two different intracellular BMP signaling pathways. In the canonical pathway, BMP binds to a pre-assembled receptor complex composed of BMPRI and BMPRII, and BMPRII phosphorylates (specified as P) BMPRI, which then initiates intracellular phosphorylation of Smad1/5/8. The phosphorylated Smad1/5/8 then forms a complex with Smad4 and enters the nucleus to regulate transcription of target genes. In the non-canonical pathway, BMP first binds to BMPRI and then recruits BMPRII to induce ERK/p38/JNK mitogen-activated protein kinase pathways. BMP, bone morphogenetic protein; BMPRI, bone morphogenetic protein type I receptor; BMPRII, bone morphogenetic protein type II receptor; ERK, extracellular signal-regulated kinase; JNK, c-Jun N-terminal kinase.

ALK3-mediated BMP signaling is crucial for the development of mineralized tissues. BMPs bind to transmembrane receptors and activate signal transduction through two pathways: the canonical Smad-dependent pathway and non-canonical Smad-independent pathways, which eventually affect gene expression in the nucleus (Figure 1). The activation of the Smad pathway or the Smad-independent pathway is determined by the hetero-oligomerization pattern of cell surface receptors. The canonical Smad pathway is triggered by BMPs binding to pre-assembled type I-II receptor complex, whereas the Smad-independent pathway is initiated after BMPs first bind to high-affinity BMPRI and subsequently recruit BMPRII to form a complex (Nohe et al., 2002). During canonical Smad-dependent pathway, the receptor complex phosphorylates receptor-regulated Smad (R-Smad) 1/5/8 (Szilágyi et al., 2022), and then phosphorylated R-Smads bind to common-partner Smad (Co-Smad) 4 to form multimeric protein complexes which translocate into the nucleus to regulate gene expression. In addition to canonical pathway, BMPs can also conduct signal transduction through non-canonical Smad-independent pathways, including extracellular signal-regulated kinase, c-Jun N-terminal kinase, and p38 mitogen-activated protein kinase pathways (Miyazono et al., 2005; Gomez-Puerto et al., 2019).

BMP signaling also exhibits crosstalk with other signaling pathways. First of all, BMP signaling has a dual role in regulating Wnt signaling (Wu et al., 2016): it can inhibit Wnt/β-catenin signaling by upregulating the expression of Wnt antagonists Dickkopf-1 (DKK1) and sclerostin (SOST) and preventing β-catenin nuclear translocation; and it can also promote Wnt/β-catenin signaling by upregulating Wnt expression and forming a co-transcriptional complex with β-catenin/Runt-related transcription factor 2 (RUNX2). Second, BMP signaling interacts with Indian hedgehog/parathyroid hormone-related protein signal axis (Wang et al., 2017).

ALK3, a transmembrane receptor protein, is extensively expressed in various tissues and regulates early embryonic and tissue development. In mice with ALK3 knockout induced by tamoxifen at different ages, an increase in trabecular bone volume was observed at the weaning and adult stages; however, this change differed in different parts of the bone (Kamiya et al., 2008). Furthermore, knocking out ALK3 in the craniofacial primordium of Nestin-Cre; ALK3^f/f^ mouse embryos led to bilateral cleft lip and palate, and arrest of maxillary molar tooth germ development (Liu et al., 2005). Therefore, ALK3 has great potential in the formation and repair of mineralized hard tissues.

## 3 ALK3 in the formation of dental mineralized tissues

Tooth development is initiated by a series of interactions between epithelial and mesenchymal stem cells (MSCs). The mesenchyme condenses around the epithelial tooth bud and, via the expression of a particular set of transcription factors and signaling molecules, gains the ability to instruct tooth morphogenesis (Li et al., 2017). The tooth bud consists of three parts: the enamel organ, responsible for enamel formation; the dental papilla, which generates dentin and pulp; and the dental follicle, which forms cementum, the periodontal ligament, and part of the alveolar bone. The development of the enamel organ is a consecutive process that includes the bud, cap, and bell stages. During the bell stage, the inner enamel epithelium stimulates MSCs within the dental papilla to undergo differentiation into odontoblasts, while these epithelial cells themselves begin to differentiate into ameloblasts. Li et al. demonstrated that neural crest-specific disruption of ALK3 resulted in the impaired differentiation of dental mesenchymal component, thereby impeding the development of bud or cap stage tooth germs (Li et al., 2011). ALK3 plays critical roles in regulating the formation of dental mineralized tissues.

### 3.1 ALK3 in tooth enamel formation

**FIGURE 2:**
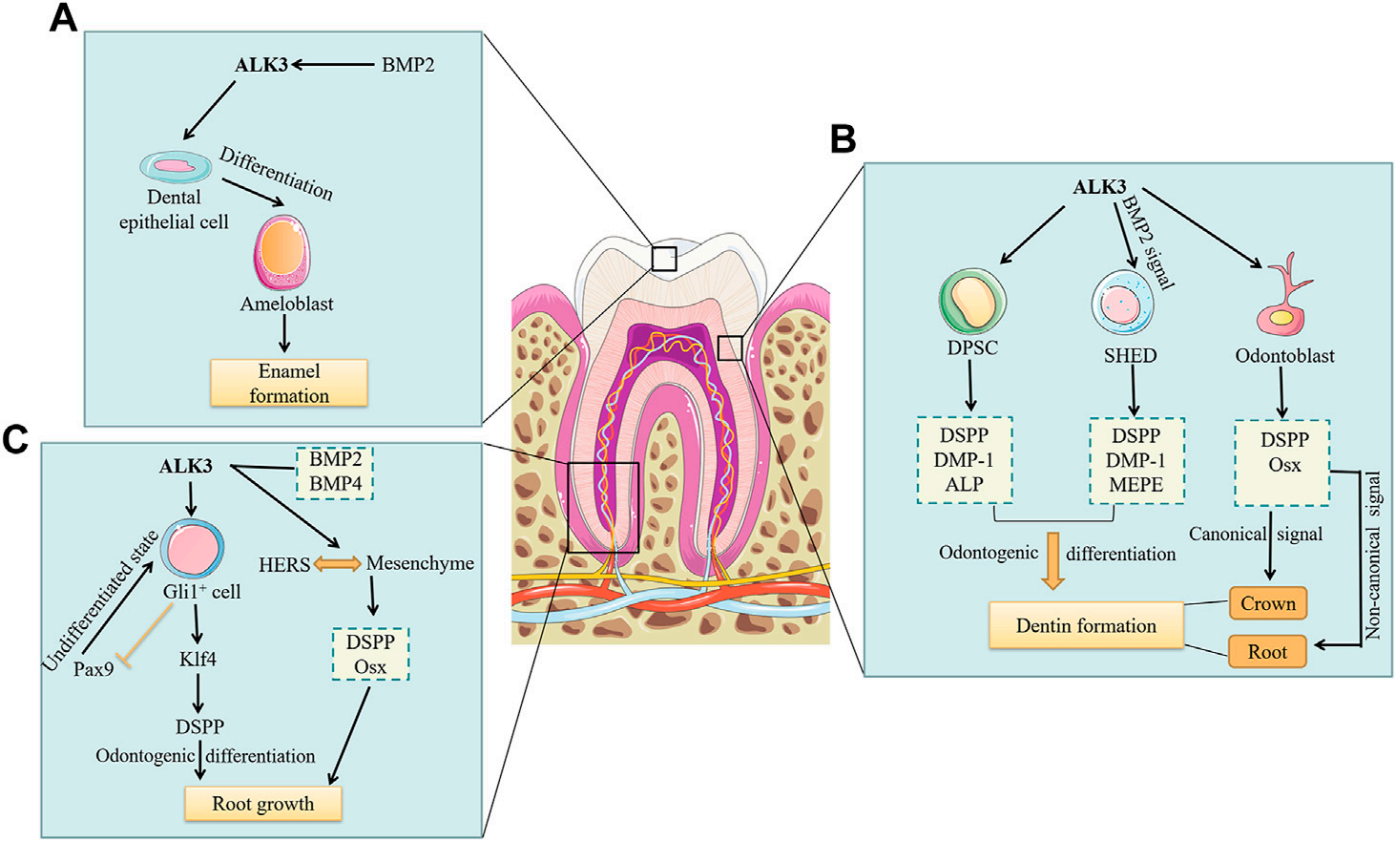
The roles of ALK3 in the formation of dental mineralized tissues. Current studies on the roles of ALK3 in dental mineralized tissue formation have only included the roles of ALK3 in enamel, dentin, and root formation. **(A)** BMP2 binds to the high-affinity ALK3, which in turn promotes the differentiation of dental epithelial cells into ameloblasts. Ameloblasts ultimately promote enamel formation. **(B)** ALK3 promotes the odontogenic differentiation of DPSCs by upregulating the expression of DSPP, DMP-1 and ALP, ultimately leading to dentin formation. Similarly, ALK3-mediated BMP2 signaling promotes odontogenic differentiation of SHEDs by upregulating the expression of DSPP, DMP-1 and MEPE, ultimately leading to dentin formation. While ALK3 in odontoblasts promotes the expression of DSPP and Osx. ALK3-mediated canonical BMP signaling in odontoblasts controls crown dentin formation, whereas non-canonical BMP signaling may regulate root dentin formation. **(C)** ALK3 in Gli^+^ cells promotes Klf4 expression, which in turn activates DSPP to promote odontogenic differentiation and ultimately root growth. Meanwhile, ALK3 restricts the expression of Pax9 to facilitate the maturation and differentiation of Gli^+^ cells. ALK3-mediated BMP2 and BMP4 signaling can maintain the interaction between HERS and root mesenchyme and promote DSPP and Osx expression in root mesenchyme to induce root growth. BMP, bone morphogenetic protein; ALK3, activin receptor-like kinase 3; DPSC, dental pulp stem cell; SHED, stem cell from human exfoliated deciduous teeth; DSPP, dentin sialophosphoprotein; DMP-1, dentin matrix protein-1; ALP, alkaline phosphatase; MEPE, matrix extracellular phosphoglycoprotein; Pax9, paired box gene 9; Klf4, kruppel-like factor 4; Osx, osterix; HERS, Hertwig’s epithelial root sheath.

The formation of tooth enamel depends on the physiological activity of ameloblasts. Ameloblasts, derived from primitive dental epithelial cells, express amelogenin and form tooth enamel. Ameloblasts are shed by apoptosis during enamel maturation and tooth eruption. It have been found that Wnt1-Cre; ALK3^f/−^ mice showed ectopic cementum-like structures and termination of the differentiation process of dental epithelial cells, which in turn disrupted ameloblast formation (Li et al., 2011). Similarly, inhibition of ALK3-mediated BMP signaling in mice resulted in impaired ameloblast differentiation, reduced amelogenin expression, and defective enamel formation (Cao et al., 2013; Miao et al., 2022). BMP2, a ligand of the BMP signaling pathway, has a high affinity for ALK3. Yang et al. obtained BMP2-cKO^od^ mutant mice by crossing BMP2^f/f^ with 3.6Col1a1-Cre mice (Yang et al., 2012). The authors observed an 80% decrease in phosphorylated Smad1/5/8 levels in odontoblasts and ameloblasts of the 1-day-old knockout mice compared to control group. In addition, in the five-day-old mutant mice, the expression of dentin sialophosphoprotein (DSPP) decreased by 90% and the ameloblasts had normal morphology but delayed initial enamel formation. Therefore, BMP2 is important for both dentin and enamel formation, but the signaling mechanisms involved were not mentioned in this study. In summary, the research outlined here demonstrates that ALK3 and its ligand, BMP2, both are necessary for the differentiation of dental epithelial cells into ameloblasts and further affect tooth enamel formation (Figure 2A).

### 3.2 ALK3 in dentin formation

Dental MSCs are essential for dentin formation. The quiescent Gli1^+^ cells located near the neurovascular bundle are considered to be typical MSCs. Shi et al. (2019) demonstrated that Gli1-Cre^ERT2^; ALK3^f/f^ mice had central incisors with shortened dentin, and an absence of DSPP from incisor proximal regions, suggesting that ALK3 in Gli1^+^ MSCs affects cell fate and dentin formation. Dental pulp stem cells (DPSCs) and stem cells from human exfoliated deciduous teeth (SHEDs) are two types of odontogenic MSCs. Zhu et al. found that the expression of ALK3 and the phosphorylation level of Smad5 both increased in a dose-dependent manner in DPSCs induced by BMP7 for 7 and 14 days (Zhu et al., 2018). Furthermore, DSPP, dentin matrix protein-1 (DMP-1), and alkaline phosphatase (ALP) were also upregulated, indicating that BMP7 promotes odontogenic differentiation of DPSCs through the ALK3-Smad5 pathway. Casagrande et al. observed that SHEDs highly expressed BMP receptors, but the expression of DSPP, DMP-1, and matrix extracellular phosphoglycoprotein (MEPE), three odontoblast differentiation markers, almost disappeared after blocking BMP2 by neutralizing antibodies in SHEDs (Casagrande et al., 2010). These findings suggest that ALK3-mediated BMP signaling plays vital roles in the odontogenic differentiation of MSCs (Figure 2B).

Besides the influence of stem cells, the formation of dentin mainly depends on the physiological activity of odontoblasts. Odontoblasts are mesenchyme-derived cells, with cell bodies arranged in the dentin wall proximal to the pulp. Their protrusions extend into the dentin tubules to the enamel-dentinal junction (Shuhaibar et al., 2021). In addition to forming dentin, odontoblasts sense nociceptive pain (Wen et al., 2017) and cold stimuli (Tazawa et al., 2017), act as surveillance cells to detect invading pathogens and trigger immune responses (Yumoto et al., 2018). DSPP is crucial in odontoblasts, serving as a marker of terminal differentiation. Studies have shown that ALK3-mediated BMP signaling controls odontoblast differentiation by regulating DSPP expression (Li et al., 2011; Parsegian, 2023). Omi et al. used Osx-Cre to conditionally knock out ALK3 in mouse odontoblasts, resulting in postnatal dentin thickness reduction, molar root shortening, and downregulation of Osx and DSPP expression (Omi et al., 2020). Constitutive activation of ALK3 rescued Smad 1/5/9 activity and crown dentin formation in ALK3 cKO mice, whereas impaired root dentin formation and expression of Osx and DSPP were unchanged. Therefore, their findings suggest that crown and root odontoblasts are heterogeneous, i.e., ALK3-mediated canonical BMP signaling in odontoblasts controls crown dentin formation, whereas non-canonical BMP signaling may regulate root dentin formation (Figure 2B). The specific molecular mechanisms of these two signals in dentin formation at different tooth sites require further exploration.

It is worth noting that the main part of the tooth root consists of dentin, and therefore the root formation is closely related to the dentin formation. Feng et al. identified the MSC cell population supporting molar root growth as Gli1^+^ cells and found that in Gli1-CreER; ALK3^f/f^ mice, the molar roots were missing, and paired box gene 9 (Pax9) was upregulated while kruppel-like factor 4 (Klf4) was reduced (Feng et al., 2017). Klf4 can significantly activate DSPP to promote Gli1^+^ MSCs odontogenic differentiation, while Pax9 is critical for maintaining Gli1^+^ MSCs in an undifferentiated state (Feng et al., 2017). Therefore, ALK3 in Gli1^+^ MSCs promotes odontogenic differentiation by restricting Pax9 expression and promoting Klf4 expression. In addition, Hertwig’s epithelial root sheath (HERS) functions as a signal center that induces root formation and critically affects the number, shape, and length of tooth roots (Li et al., 2017). But the prolonged presence of HERS also prevents root formation. Mu et al. found that in K14-Cre; Bmp2^f/f^;Bmp4^f/f^ mice, HERS persisted and the interaction between HERS and root mesenchyme was impaired, which in turn downregulated the expression of Osx and DSPP in the root mesenchyme, hindered root dentin formation, and ultimately led to root shortening (Mu et al., 2021). In conclusion, ALK3-mediated BMP signaling not only regulates the odontogenic differentiation of dental MSCs and odontoblasts but also controls HERS degeneration at the appropriate time, affecting dentin formation and root phenotype (Figure 2C).

### 3.3 ALK3 in cementum and root formation

While there is currently a lack of direct evidence to prove the relationship between ALK3 and cementum formation, some researches show that BMPs are associated with cementum formation and root development. A study showed significantly more new bone and cementum, and less connective tissue in defects implanted with BMP-7 loaded hydrogels compared with hydrogels without BMP-7 (Zang et al., 2019). The hybrid periodontal regenerative method of calcium phosphate cement (CPC)/bone morphogenetic protein (BMP)-2/propylene glycol alginate (PGA)/fibroblast growth factor (FGF)-2 promoted periodontal regeneration, including epithelial downgrowth, cementum, and ligament regeneration (B. Wang et al., 2019). In addition, a study showed that the double abrogation of Bmp2 and Bmp4 from mouse epithelium could exhibit a persistent Hertwig’s Epithelial Root Sheath (HERS) and lead to short root anomaly (SRA), which suggested that ectoderm-derived Bmp2 and Bmp4 were critical to maintaining the epithelial-mesenchymal interaction during tooth root development, which was required for the degeneration of HERS, as well as the differentiation and maturation of root odontoblasts. In addition, several groups have discussed the role epithelial-mesenchymal interactions and molecules such as Gli1, Wnt/b-catenin, Axin 2, Nfc1 and more (Wang et al., 2022; Lav et al., 2023) during cementogenesis. However, there is currently a lack of direct evidence to prove the relationship between ALK3 and root development.

## 4 ALK3 in regulating the dynamic balance of tooth movement

Tooth movement occurs commonly during various dental events, such as tooth eruption, tooth loss, adaptation to mastication, and orthodontic tooth movement (OTM) (Yan et al., 2020). This section focuses on the regulatory roles of ALK3 in tooth eruption and OTM.

**FIGURE 3:**
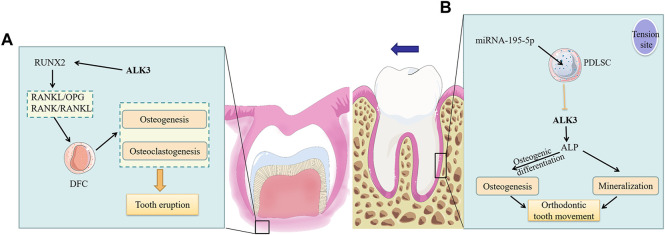
The roles of ALK3 in tooth movement. **(A)** ALK3 activates the expression of the downstream gene RUNX2 and further upregulates RANKL/OPG and RANK/RANKL in DFCs to promote osteogenesis and osteoclastogenesis, which ultimately facilitates tooth eruption by providing the required biological force and pathway. **(B)** At the tension site of periodontal ligament, ALK3 in PDLSCs stimulates ALP expression, thus promoting osteogenic differentiation of PDLSCs, increasing osteogenesis and mineralization at this site and finally facilitating OTM. While the mechanosensitive miRNA-195-5p can suppress the above effects by inhibiting ALK3. ALK3, activin receptor-like kinase 3; RUNX2, runt-related transcription factor 2; RANKL, receptor activator of nuclear factor-κB ligand; RANK, receptor activator of nuclear factor-κB; OPG, osteoprotegerin; DFC, dental follicle cell; PDLSC, periodontal ligament stem cell; ALP, alkaline phosphatase.

During tooth eruption, alveolar bone undergoes constant remodeling with a high turnover rate of bone formation and bone resorption. Jaw bone osteoblasts increase alveolar bone volume and length by promoting osteogenesis around the radicular portion of the dental follicle, which generates the biological force necessary for tooth eruption (Isawa et al., 2019; Xin et al., 2022). Dental follicle cells (DFCs), as stem cells present in the dental follicle tissue, are direct progenitor cells of the periodontal tissue and can differentiate into different types of cells necessary for tooth eruption. RUNX2, a critical downstream target of the BMP pathway (Liu et al., 2022), can regulate osteoclasts to form the tooth eruption pathway (Xin et al., 2022). The RUNX2 mutation in mice impaired receptor activator of nuclear factor-κB ligand (RANKL)/osteoprotegerin (OPG) and receptor activator of nuclear factor-κB (RANK)/RANKL signaling in DFCs during osteoclastogenesis, and negatively affected osteogenesis, resulting in arrested tooth eruption (Zeng et al., 2022). Studies have demonstrated that the inhibition of endogenous ALK3 inhibits the RANKL/RANK/OPG signaling pathway (Geng et al., 2019; Wang et al., 2020). In DFCs, RANKL and OPG are both expressed (Uribe et al., 2018), and play key roles in tooth eruption (Brodetska et al., 2020). Therefore, it can be inferred that ALK3 in DFCs promotes tooth eruption by upregulating RUNX2 and RANKL/OPG (Figure 3A). However, this hypothesis needs further experimental validation.

The biological response of the periodontal ligament triggers periodontal tissue reconstruction, strongly involved in the process of OTM (Brockhaus et al., 2021). Periodontal ligament stem cells (PDLSCs), which are incompletely differentiated MSCs located in the periodontal ligament, have self-renewal ability, multipotency, and immunomodulatory properties (Huang et al., 2018). Further, they maintain periodontal tissue homeostasis. OTM is accomplished through the dynamic remodeling of the periodontal ligament and alveolar bone under mechanical loading. In this process, PDLSCs differentiate into osteoblasts in areas of tension and short-term compression, leading to bone deposition; conversely, they differentiate into osteoclasts in areas of long-term compression, leading to bone resorption (Huang et al., 2018). Chang et al. demonstrated that ALK3 was a direct target of mechanosensitive miRNA-195-5p, which could inhibit the osteogenic differentiation of PDLSCs by suppressing ALK3 translation (Chang et al., 2017). Supporting this, reintroduction of ALK3 during miR-195-5p overexpression *in vitro* upregulated ALP, thus restoring osteogenic activity of PDLSCs. Furthermore, mechanical loading at tension sites downregulated miR-195-5p while ALK3 was upregulated, which promoted osteogenic differentiation and mineralization (Figure 3B). This study demonstrated that ALK3 rescued inhibited osteogenic differentiation of PDLSCs. Therefore, ALK3 could be useful in clinical applications for alleviating adverse effects such as periodontal disease and alveolar bone resorption caused by orthodontic treatment. In the process of OTM, not only periodontal ligament and alveolar bone but also cementum are changed. Researches showed that in the process of OTM, high-dose rhBMP2 induces root resorption, while low-dose rhBMP2 causes only partial cementum resorption on the pressure side (Kawamoto et al., 2003). And high levels of BMP-2 in the periodontal tissue of orthodontic teeth can activate osteoclasts and cementoclasts, causing aggressive root resorption (Wang et al., 2023).

## 5 Regulation of ALK3 in bone remodeling

The main cells involved in bone remodeling include osteoblasts, osteoclasts, and osteocytes. Bone homeostasis is maintained through a balance between the physiological functions of osteoblast and osteoclast. Osteocytes are multifunctional cells with many key regulatory roles in bone tissue, including acting as mechanosensory and endocrine cells and controlling bone remodeling by regulating both osteoclasts and osteoblasts. It is well known that ALK3 plays a critical role in osteogenesis (Mang et al., 2020; Li et al., 2021). Therefore, it is essential to further understand the specific regulatory mechanism of ALK3 in the above cells.

### 5.1 Osteoblasts

ALK3 is vitally involved in bone formation and remodeling by limiting osteoblast proliferation while promoting differentiation (Lim et al., 2016). Bone strength is affected by bone mass and bone quality. Bao et al. found that in osteoblast ALK3-specific knockout mice, the bone mass of long bones was increased, while bone quality was significantly decreased, which led to the decreased bone strength (Bao et al., 2018). Moreover, the expression of osteocalcin, an osteoblast terminal differentiation marker, was decreased, indicating that ALK3 loss impaired osteoblast differentiation. SOST and DKK1 inhibit Wnt/β-catenin signaling, thereby regulating bone mass. Kamiya et al. demonstrated that osteoblast ALK3 cKO in mice downregulated downstream SOST and DKK1, resulting in increased Wnt/β-catenin signaling and bone mass (Kamiya et al., 2010). At the same time, DKK1 downregulation also impeded osteoblast terminal differentiation, inhibited mineralization, and decreased bone strength (Kamiya et al., 2010; Bao et al., 2018). These findings illustrate that ALK3 knockout in osteoblasts can increase bone mass while significantly reduce bone quality at the same time, thus turning out decreased bone strength. Supporting this, the BMP inhibitor Noggin can also downregulate SOST and DKK1, thereby activating canonical Wnt signaling, which in turn affects postnatal skeletal development (Kamiya et al., 2010). In conclusion, ALK3 in osteoblasts can control bone mass through negatively regulating Wnt/β-catenin signaling pathway by SOST and DKK1 while maintain bone strength by promoting osteoblast differentiation. ALK3 mediates the crosstalk between BMP signaling and Wnt signaling to maintain normal bone function. However, downregulation of Wnt signaling mediated by DKK1 at least partially lead to osteolytic lesions (Li et al., 2006). And loss-of-function mutations in the SOST gene lead to sclerosteosis. (Sebastian and Loots, 2018).

Studies have investigated the mechanism of increased bone mass following osteoblast ALK3 loss. After ALK3 (Og2-Cre) cKO, Mishina et al. found that young mutant mice exhibited lower bone mass because of reduced bone formation, while older mutant mice had higher bone mass owing to significantly decreased bone resorption (Mishina et al., 2004). Another study showed that bone formation markers RUNX2 and bone sialoprotein were significantly decreased in adult vertebrae of osteoblast ALK3-specific knockout mice, while ALP2 and Osx were unchanged, and the bone resorption markers tartrate-resistant acid phosphatase and matrix metallopeptidase 9 were significantly decreased (Kamiya et al., 2008). In contrast, while tartrate-resistant acid phosphatase was significantly reduced, the bone formation markers in weanling mouse ribs remained unchanged (Kamiya et al., 2008). The results indicated that the decrease in bone resorption in mutant mice was significantly greater than that in bone formation, resulting in increased bone mass. The summarized studies mainly attributed bone mass increase to inhibited bone resorption, i.e., impaired osteoclast formation or function. However, other work has shown that osteoclast number and activity was unchanged after osteoblast ALK3 knockout, and that bone mass increase was largely due to a rise in osteogenic precursor cells (Lim et al., 2016; Zhang et al., 2020). Consequently, the cellular and molecular mechanisms underlying the increased bone mass resulting from impaired osteoblast ALK3 signaling need to be further investigated.

### 5.2 Osteoclasts

Osteoclasts, multinucleated giant cells, are formed by the fusion of mononuclear macrophages that differentiate from myeloid progenitor cells in the bone marrow. Osteoclast function opposes that of osteoblasts; osteoclasts maintain the balance of bone remodeling through bone resorption. Okamoto et al. performed ALK3 cKO in osteoclasts and found increased expression of bone resorption markers and thickening of trabecular bone in the tibia and femur (Okamoto et al., 2011), demonstrating that ALK3 in osteoclasts negatively regulates differentiation of osteoclasts and osteoblast-mediated bone formation. Thus, ALK3 in osteoclasts modulates osteoclast-osteoblast coupling by downregulating bone formation. On the other hand, osteoblast ALK3 acts as a counter-balance in coupling: studies have shown that ALK3 inactivation decreased RANKL while increased OPG, resulting in osteoclastogenesis inhibition (Kamiya et al., 2008; Okamoto et al., 2011; Zhang et al., 2020). And decreased RANKL/OPG is accomplished through upregulation of Wnt/β-catenin signaling (Lin et al., 2016). Thus, ALK3 in osteoblasts activates DKK1 and SOST to inhibit Wnt signaling, and further upregulates RANKL/OPG to promote osteoclastogenesis. In parallel, ALK3 in osteoclasts downregulates osteoblast activity and further inhibits bone formation (Lin et al., 2016). Such findings reveal how osteoclasts interact with osteoblasts through ALK3, which mediates both bone formation and resorption. The balance between these two processes determines bone mass and its disruption by ALK3 over- or down-expression can contribute to the progression of bone-related diseases. Taken together, the osteoclast-osteoblast balance determines the bone remodeling.

### 5.3 Osteocytes

Osteocytes are flat, oval cells that differentiate from osteoblasts, with multiple protrusions into the bone canaliculi. This abundance of dendritic processes makes osteocytes the primary mechanosensing cells of the bone (Lewis et al., 2021). ALK3-mediated BMP signaling is crucial to the coupling of osteocytes to osteoblasts and to osteoclasts. In the process of bone remodeling, the paracrine signaling from osteocytes to osteoclasts plays a more prominent role compared to that from osteoblasts to osteoclasts (Nakashima et al., 2011). Specifically, the paracrine effects of osteocyte RANKL on bone remodeling are greater than those of osteoblast RANKL. Kamiya et al. conditionally knocked out ALK3 in osteocytes by DMP1-Cre, resulting in SOST and RANKL mRNA downregulation and enhanced Wnt signaling, which resulted in impaired osteocyte maturation and increased proliferation (Kamiya et al., 2016). Increased Wnt/β-catenin signaling turned out a substantially increased number of immature osteocytes, poor mineralization, and increased bone mass, while decreased RANKL/OPG resulted in the downregulation of osteoclastogenesis (Kamiya et al., 2016). In summary, ALK3 in osteocytes can affect osteoclasts through RANKL/OPG paracrine signaling and can also affect bone mass and bone quality in concert with osteoblasts through crosstalk with Wnt signaling. Therefore, the regulatory role of ALK3 in osteocytes is key for bone remodeling. However, there are fewer studies on ALK3-mediated regulation in osteocytes than in osteoblasts; thus, more osteocyte-specific studies are needed in the future.

## 6 Outlook

Years of research have demonstrated the crucial roles of ALK3-mediated BMP signaling in the development and homeostasis of teeth and bones. Gaining insight into the mechanisms underlying tooth formation, tooth movement and bone remodeling is critical for guiding clinical strategies for repairing functional mineralized tissues. Currently, tissue engineering techniques for bone and tooth repair and regeneration are widely studied, but there are still some challenges in repairing critical bone defects and regenerating dental mineralized tissues such as enamel and tubular dentin. Van Houdt et al. (2021) applied self-assembled hydrogels loaded with BMP2 to critical bone defects in rats and found significant spongy bone formation in the hydrogel group loaded with low doses of BMP2, while high doses of BMP2 resulted in cyst-like bony shells filled with adipose tissue in the defect area. The low-dose BMP2 group also had more adipose cells in the defect bridge than the control group (no BMP2 application). Thus, in addition to potentially leading to inflammation, ectopic bone, and osteoclast activation (Gillman and Jayasuriya, 2021), the use of BMP2 for bone regeneration is also related to induce adipogenic differentiation, resulting in decreased bone quality. Enamel regeneration and its potential future clinical implementation remain a daunting task. This process involves three main types of difficulties, i) new enamel no longer forms after tooth eruption, ii) it is difficult to simulate the high ion concentration and significant pH change in the initial enamel formation process, and iii) the enamel rod arrangement structure is difficult to be restored by chemical materials (Pandya and Diekwisch, 2019). Most of the current studies have utilized biological scaffolds combined with growth factors loaded or unloaded with stem cells to regenerating the pulp-dentin complex, but regeneration of functional dentin is also challenging due to the presence of dentinal tubules and odontoblastic processes. In summary, more effective repair and regeneration therapies based on the ALK3-mediated BMP signaling pathway need to be explored.

**FIGURE 4:**
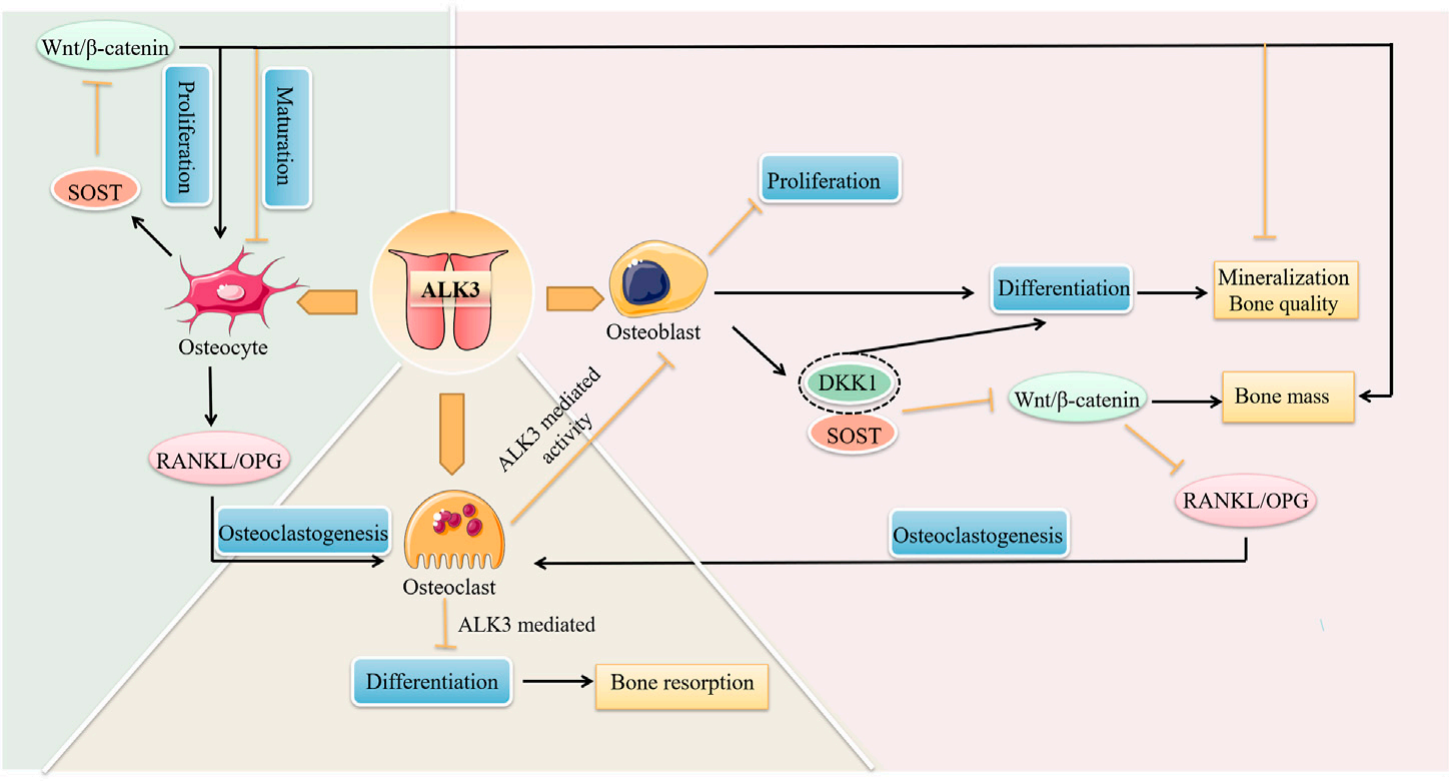
The roles of ALK3 in bone remodeling. ALK3 controls cell proliferation, differentiation, and other biological functions by different mechanisms in bone cells, and connects osteoblasts, osteoclasts, and osteocytes to form a cellular network that jointly regulates bone remodeling. ALK3, activin receptor-like kinase 3; RANKL, receptor activator of nuclear factor-κB ligand; OPG, osteoprotegerin; DKK1, dickkopf-1; SOST, sclerostin.

ALK3 not only regulates tooth formation and tooth movement, but also regulates bone remodeling by affecting the network of osteoblasts, osteoclasts, and osteocytes (Figure 4). ALK3 regulates osteoblast-osteoclast and osteocyte-osteoclast coupling by affecting RANKL/OPG. Further, ALK3 downregulates bone mass and promotes mineralization in osteoblasts and osteocytes by antagonizing Wnt/β-catenin signaling. In conclusion, ALK3 couples osteoblasts, osteoblasts, and osteoclasts to jointly regulate bone growth, bone mass, bone quality, and other skeletal characteristics. However, it is hypothesized that exaggerated activation of the BMP signaling pathway can potentially induce heterotopic ossification. This can be attenuated through use of the BMP ligand trap ALK3-Fc (Strong et al., 2021). The results of another study demonstrated that knocking down ALK3 by tamoxifen did not prevent heterotopic ossification (Agarwal et al., 2017). Therefore, the role of ALK3 in heterotopic ossification has not been fully elucidated and deserves more experimental exploration owing to contradictory results reported. Wang et al. demonstrated that a soluble form of ALK3 fusion protein (mALK3-mFc), an ALK3 antagonist, could promote osteoblastogenesis and reduce osteoclastogenesis by activating Wnt/β-catenin signaling and inhibiting RANKL/RANK/OPG signaling, respectively (Wang et al., 2020). Therefore, there is evidence for the application of mBMPR1A-mFc as a therapeutic treatment for radiation-induced osteoporosis or decreased estrogen in clinical practice. In addition to overactivation, the deletion or mutation of ALK3 can also trigger bone-related diseases. Russell et al. reported a case of a patient with a homozygous missense variant (ALK3^R406L^) that caused severe skeletal abnormalities, facial dysmorphisms, and developmental delays (Russell et al., 2019).

Based on the current knowledge, the regulatory mechanisms of ALK3 on mineralized tissues are relatively clear. However, most studies of mutant animals have simply highlighted tooth developmental defect phenotypes but have yet to elucidate the molecular regulatory network. Thus, more and deeper studies are needed. The findings in this review provide a direction for future ALK3-based studies. Since ALK3 receives many different signals, altering this receptor carries the potential for unintended side effects; this will require experimental investigation. In addition, existing studies have some inconsistent conclusions that can be caused by different experimental animals, anatomical sites, and *in vitro*/vivo environments. In conclusion, more basic research is required before addressing new clinical treatment designs based on ALK3-mediated BMP signaling.

## References

[B1] Abou Neel E. A. Aljabo A. Strange A. Ibrahim S. Coathup M. Young A. M. (2016). Demineralization-remineralization dynamics in teeth and bone. Int. J. Nanomed. 11, 4743–4763. 10.2147/IJN.S107624 PMC5034904 27695330

[B2] Agarwal S. Loder S. J. Breuler C. Li J. Cholok D. Brownley C. (2017). Strategic targeting of multiple BMP receptors prevents trauma-induced heterotopic ossification. Mol. Ther. 25, 1974–1987. 10.1016/j.ymthe.2017.01.008 28716575 PMC5542633

[B3] Aoki H. Fujii M. Imamura T. Yagi K. Takehara K. Kato M. (2001). Synergistic effects of different bone morphogenetic protein type I receptors on alkaline phosphatase induction. J. Cell Sci. 114, 1483–1489. 10.1242/jcs.114.8.1483 11282024

[B4] Bakopoulou A. Papachristou E. Bousnaki M. Hadjichristou C. Kontonasaki E. Theocharidou A. (2016). Human treated dentin matrices combined with Zn-doped, Mg-based bioceramic scaffolds and human dental pulp stem cells towards targeted dentin regeneration. Dent. Mater 32, e159–e175. 10.1016/j.dental.2016.05.013 27298239

[B5] Balic A. (2018). Biology explaining tooth repair and regeneration: A mini-review. Gerontology 64, 382–388. 10.1159/000486592 29533942

[B6] Bao Q. Li A. Chen S. Feng J. Liu H. Qin H. (2018). Disruption of bone morphogenetic protein type IA receptor in osteoblasts impairs bone quality and bone strength in mice. Cell Tissue Res. 374, 263–273. 10.1007/s00441-018-2873-3 29987355

[B7] Brockhaus J. Craveiro R. B. Azraq I. Niederau C. Schröder S. K. Weiskirchen R. (2021). *In vitro* compression model for orthodontic tooth movement modulates human periodontal ligament fibroblast proliferation, apoptosis and cell cycle. Biomolecules 11, 932. 10.3390/biom11070932 34201602 PMC8301966

[B8] Brodetska L. Natrus L. Lisakovska O. Kaniura O. Iakovenko L. Skrypnyk I. (2020). The regulatory role of the RANKL/RANK/OPG signaling pathway in the mechanisms of tooth eruption in patients with impacted teeth. BMC oral health 20, 261. 10.1186/s12903-020-01251-y 32948158 PMC7501598

[B9] Cao H. Jheon A. Li X. Sun Z. Wang J. Florez S. (2013). The Pitx2:miR-200c/141:noggin pathway regulates Bmp signaling and ameloblast differentiation. Development 140, 3348–3359. 10.1242/dev.089193 23863486 PMC3737717

[B10] Casagrande L. Demarco F. F. Zhang Z. Araujo F. B. Shi S. Nör J. E. (2010). Dentin-derived BMP-2 and odontoblast differentiation. J. Dent. Res. 89, 603–608. 10.1177/0022034510364487 20351355

[B11] Chang M. L. Lin H. Fu H. D. Wang B. K. Han G. L. Fan M. W. (2017). MicroRNA-195-5p regulates osteogenic differentiation of periodontal ligament cells under mechanical loading. J. Cell Physiol. 232, 3762–3774. 10.1002/jcp.25856 28181691

[B12] Chen X. Tan B. Bao Z. Wang S. Tang R. Wang Z. (2021). Enhanced bone regeneration via spatiotemporal and controlled delivery of a genetically engineered BMP-2 in a composite Hydrogel. Biomaterials 277, 121117. 10.1016/j.biomaterials.2021.121117 34517277

[B13] Corps K. Stanwick M. Rectenwald J. Kruggel A. Peters S. B. (2021). Skeletal deformities in osterix-Cre;Tgfbr2^f/f^ mice may cause postnatal death. Genes 12, 975. 10.3390/genes12070975 34202311 PMC8307487

[B14] Duan P. Bonewald L. F. (2016). The role of the wnt/β-catenin signaling pathway in formation and maintenance of bone and teeth. Int. J. Biochem. Cell Biol. 77, 23–29. 10.1016/j.biocel.2016.05.015 27210503 PMC4958569

[B15] Feng J. F. Jing J. J. Li J. Y. Zhao H. Punj V. Zhang T. W. (2017). BMP signaling orchestrates a transcriptional network to control the fate of mesenchymal stem cells in mice. Development 144, 2560–2569. 10.1242/dev.150136 28576771 PMC5536932

[B16] Geng Q. Heng K. Li J. Wang S. Sun H. Sha L. (2019). A soluble bone morphogenetic protein type 1A receptor fusion protein treatment prevents glucocorticoid-Induced bone loss in mice. Am. J. Transl. Res. 11, 4232–4247. 31396331 PMC6684880

[B17] Gillman C. E. Jayasuriya A. C. (2021). FDA-approved bone grafts and bone graft substitute devices in bone regeneration. Mat. Sci. Eng. C-mater. 130, 112466. 10.1016/j.msec.2021.112466 PMC8555702 34702541

[B18] Glass D. A. Bialek P. Ahn J. D. Starbuck M. Patel M. S. Clevers H. (2005). Canonical Wnt signaling in differentiated osteoblasts controls osteoclast differentiation. Dev. Cell. 8, 751–764. 10.1016/j.devcel.2005.02.017 15866165

[B19] Gomez-Puerto M. C. Iyengar P. V. García de Vinuesa A. Ten Dijke P. Sanchez-Duffhues G. (2019). Bone morphogenetic protein receptor signal transduction in human disease. J. Pathol. 247, 9–20. 10.1002/path.5170 30246251 PMC6587955

[B20] Heldin C. H. Moustakas A. (2016). Signaling receptors for TGF-β family members. Cold Spring Harb. Perspect. Biol. 8, a022053. 10.1101/cshperspect.a022053 27481709 PMC4968163

[B21] Huang H. Yang R. Zhou Y. H. (2018). Mechanobiology of periodontal ligament stem cells in orthodontic tooth movement. Stem Cells Int. 2018, 6531216. 10.1155/2018/6531216 30305820 PMC6166363

[B22] Isawa M. Karakawa A. Sakai N. Nishina S. Kuritani M. Chatani M. (2019). Biological effects of anti-RANKL antibody and zoledronic acid on growth and tooth eruption in growing mice. Sci. Rep. 9, 19895. 10.1038/s41598-019-56151-1 31882595 PMC6934544

[B23] Kamiya N. Kobayashi T. Mochida Y. Yu P. B. Yamauchi M. Kronenberg H. M. (2010). Wnt inhibitors Dkk1 and Sost are downstream targets of BMP signaling through the type IA receptor (BMPRIA) in osteoblasts. J. Bone Min. Res. 25, 200–210. 10.1359/jbmr.090806 PMC3153381 19874086

[B24] Kamiya N. Shuxian L. Yamaguchi R. Phipps M. Aruwajoye O. Adapala N. S. (2016). Targeted disruption of BMP signaling through type IA receptor (BMPR1A) in osteocyte suppresses SOST and RANKL, leading to dramatic increase in bone mass, bone mineral density and mechanical strength. Bone 91, 53–63. 10.1016/j.bone.2016.07.002 27402532

[B25] Kamiya N. Ye L. Kobayashi T. Lucas D. J. Mochida Y. Yamauchi M. (2008). Disruption of BMP signaling in osteoblasts through type IA receptor (BMPRIA) increases bone mass. J. Bone Min. Res. 23, 2007–2017. 10.1359/jbmr.080809 PMC2686924 18684091

[B26] Kawamoto T. Motohashi N. Kitamura A. Baba Y. Suzuki S. Kuroda T. (2003). Experimental tooth movement into bone induced by recombinant human bone morphogenetic protein-2. Cleft Palate-Craniofacial J. Off. Publ. Am. Cleft Palate-Craniofacial Assoc. 40, 538–543. 10.1597/1545-1569_2003_040_0538_etmibi_2.0.co_2 12943432

[B27] Kessler E. Takahara K. Biniaminov L. Brusel M. Greenspan D. S. (1996). Bone morphogenetic protein-1: the type I procollagen C-proteinase. Science 271, 360–362. 10.1126/science.271.5247.360 8553073

[B28] Kim E. J. Lee M. J. Li L. Yoon K. S. Kim K. S. Jung, H.S. (2014). Failure of tooth formation mediated by miR-135a overexpression via BMP signaling. J. Dent. Res. 93, 571–575. 10.1177/0022034514529303 24667771

[B29] Komorowski M. (2022). Making sense of BMP signaling complexity. Cell Syst. 13, 349–351. 10.1016/j.cels.2022.04.002 35588697

[B30] Lav R. Krivanek J. Anthwal N. Tucker A. S. (2023). Wnt signaling from Gli1-expressing apical stem/progenitor cells is essential for the coordination of tooth root development. Stem Cell Rep. 18, 1015–1029. 10.1016/j.stemcr.2023.02.004 PMC10147554 36931279

[B31] Lewis K. J. Cabahug-Zuckerman P. Boorman-Padgett J. F. Basta-Pljakic J. Louie J. Stephen S. (2021). Estrogen depletion on *in vivo* osteocyte calcium signaling responses to mechanical loading. Bone 152, 116072. 10.1016/j.bone.2021.116072 34171514 PMC8316427

[B32] Li J. Parada C. Chai Y. (2017). Cellular and molecular mechanisms of tooth root development. Development 144, 374–384. 10.1242/dev.137216 28143844 PMC5341797

[B33] Li J. Sarosi I. Cattley R. C. Pretorius J. Asuncion F. Grisanti M. (2006). Dkk1-mediated inhibition of Wnt signaling in bone results in osteopenia. Bone 39, 754–766. 10.1016/j.bone.2006.03.017 16730481

[B34] Li L. Lin M. Wang Y. Cserjesi P. Chen Z. Chen Y. (2011). BmprIa is required in mesenchymal tissue and has limited redundant function with BmprIb in tooth and palate development. Dev. Biol. 349, 451–461. 10.1016/j.ydbio.2010.10.023 21034733 PMC3019275

[B35] Li N. Liu J. Liu H. Wang S. Hu P. Zhou H. (2021). Altered BMP-Smad4 signaling causes complete cleft palate by disturbing osteogenesis in palatal mesenchyme. J. Mol. Histol. 52, 45–61. 10.1007/s10735-020-09922-4 33159638

[B36] Lim J. Shi Y. Karner C. M. Lee S. Y. Lee W. C. He G. (2016). Dual function of Bmpr1a signaling in restricting preosteoblast proliferation and stimulating osteoblast activity in mouse. Development 143, 339–347. 10.1242/dev.126227 26657771 PMC4725340

[B37] Lin S. Svoboda K. K. Feng J. Q. Jiang X. (2016). The biological function of type I receptors of bone morphogenetic protein in bone. Bone Res. 4, 16005. 10.1038/boneres.2016.5 27088043 PMC4820739

[B38] Liu M. Goldman G. MacDougall M. Chen S. (2022). BMP signaling pathway in dentin development and diseases. Cells 11, 2216. 10.3390/cells11142216 35883659 PMC9317121

[B39] Liu W. Sun X. Braut A. Mishina Y. Behringer R. R. Mina M. (2005). Distinct functions for Bmp signaling in lip and palate fusion in mice. Development 132, 1453–1461. 10.1242/dev.01676 15716346

[B40] Machla F. Sokolova V. Platania V. Prymak O. Kostka K. Kruse B. (2023). Tissue engineering at the dentin-pulp interface using human treated dentin scaffolds conditioned with DMP1 or BMP2 plasmid DNA-carrying calcium phosphate nanoparticles. Acta Biomater. 159, 156–172. 10.1016/j.actbio.2023.01.044 36708852

[B41] Mang T. Kleinschmidt-Doerr K. Ploeger F. Schoenemann A. Lindemann S. Gigout A. (2020). BMPR1A is necessary for chondrogenesis and osteogenesis, whereas BMPR1B prevents hypertrophic differentiation. J. Cell. Sci. 133, jcs246934. 10.1242/jcs.246934 32764110

[B42] Miao X. Niibe K. Fu Y. Zhang M. Nattasit P. Ohori-Morita Y. (2022). Epiprofin transcriptional activation promotes ameloblast induction from mouse induced pluripotent stem cells *via* the BMP-smad signaling Axis. Front. Bioeng. Biotechnol. 10, 890882. 10.3389/fbioe.2022.890882 35800329 PMC9253510

[B43] Mishina Y. Starbuck M. W. Gentile M. A. Fukuda T. Kasparcova V. Seedor J. G. (2004). Bone morphogenetic protein type IA receptor signaling regulates postnatal osteoblast function and bone remodeling. J. Biol. Chem. 279, 27560–27566. 10.1074/jbc.M404222200 15090551

[B44] Miyazono K. Maeda S. Imamura T. (2005). BMP receptor signaling: Transcriptional targets, regulation of signals, and signaling cross-talk. Cytokine Growth Factor Rev. 16, 251–263. 10.1016/j.cytogfr.2005.01.009 15871923

[B45] Mu H. Liu X. Geng S. Su D. Chang H. Li L. (2021). Epithelial bone morphogenic protein 2 and 4 are indispensable for tooth development. Front. Physiol. 12, 660644. 10.3389/fphys.2021.660644 34483952 PMC8415269

[B46] Muir A. M. Ren Y. Butz D. H. Davis N. A. Blank R. D. Birk D. E. (2014). Induced ablation of Bmp1 and Tll1 produces osteogenesis imperfecta in mice. Hum. Mol. Genet. 23, 3085–3101. 10.1093/hmg/ddu013 24419319 PMC4030766

[B47] Nakashima T. Hayashi M. Fukunaga T. Kurata K. Oh-Hora M. Feng J. Q. (2011). Evidence for osteocyte regulation of bone homeostasis through RANKL expression. Nat. Med. 17, 1231–1234. 10.1038/nm.2452 21909105

[B48] Nauth A. Schemitsch E. Norris B. Nollin Z. Watson J. T. (2018). Critical-size bone defects: Is there a consensus for diagnosis and treatment? *J. Orthop. Trauma*. S7-S11 32, S7–S11. 10.1097/BOT.0000000000001115 29461395

[B49] Nohe A. Hassel S. Ehrlich M. Neubauer F. Sebald W. Henis Y. I. (2002). The mode of bone morphogenetic protein (BMP) receptor oligomerization determines different BMP-2 signaling pathways. J. Biol. Chem. 277, 5330–5338. 10.1074/jbc.M102750200 11714695

[B50] Okamoto M. Murai J. Imai Y. Ikegami D. Kamiya N. Kato S. (2011). Conditional deletion of Bmpr1a in differentiated osteoclasts increases osteoblastic bone formation, increasing volume of remodeling bone in mice. J. Bone Min. Res. 26, 2511–2522. 10.1002/jbmr.477 21786321

[B51] Omi M. Kulkarni A. K. Raichur A. Fox M. Uptergrove A. Zhang H. (2020). BMP-smad signaling regulates postnatal crown dentinogenesis in mouse molar. JBMR plus 4, e10249. 10.1002/jbm4.10249 32149267 PMC7017888

[B52] Pandya M. Diekwisch T. G. H. (2019). Enamel biomimetics-fiction or future of dentistry. Int. J. Oral Sci. 11, 8. 10.1038/s41368-018-0038-6 30610185 PMC6320371

[B53] Parsegian K. (2023). The BMP and FGF pathways reciprocally regulate odontoblast differentiation. Connect. Tissue Res. 64, 53–63. 10.1080/03008207.2022.2094789 35816114 PMC9832171

[B54] Peters S. B. Wang Y. Serra R. (2017). Tgfbr2 is required in osterix expressing cells for postnatal skeletal development. Bone 97, 54–64. 10.1016/j.bone.2016.12.017 28043895 PMC5368008

[B55] Russell B. E. Rigueur D. Weaver K. N. Sund K. Basil J. S. Hufnagel R. B. (2019). Homozygous missense variant in BMPR1A resulting in BMPR signaling disruption and syndromic features. Mol. Genet. Genomic Med. 7, e969. 10.1002/mgg3.969 31493347 PMC6825850

[B56] Salazar V. S. Gamer L. W. Rosen V. (2016). BMP signalling in skeletal development, disease and repair. Nat. Rev. Endocrinol. 12, 203–221. 10.1038/nrendo.2016.12 26893264

[B57] Sampath T. K. Rashka K. E. Doctor J. S. Tucker R. F. Hoffmann F. M. (1993). Drosophila transforming growth factor beta superfamily proteins induce endochondral bone formation in mammals. Proc. Natl. Acad. Sci. U. S. A. 90, 6004–6008. 10.1073/pnas.90.13.6004 8327474 PMC46855

[B58] Sanchez-Duffhues G. Williams E. Goumans M.-J. Heldin C.-H. ten Dijke P. (2020). Bone morphogenetic protein receptors: Structure, function and targeting by selective small molecule kinase inhibitors. Bone 138, 115472. 10.1016/j.bone.2020.115472 32522605

[B59] Sebastian A. Loots G. G. (2018). Genetics of Sost/SOST in sclerosteosis and van Buchem disease animal models. Metab. Clin. Exp. 80, 38–47. 10.1016/j.metabol.2017.10.005 29080811

[B60] Shi C. Wu T. He Y. Zhang Y. Fu D. (2020). Recent advances in bone-targeted therapy. Pharmacol. Ther. 207, 107473. 10.1016/j.pharmthera.2020.107473 31926198

[B61] Shi C. Yuan Y. Guo Y. Jing J. Ho T. V. Han X. (2019). BMP signaling in regulating mesenchymal stem cells in incisor homeostasis. J. Dent. Res. 98, 904–911. 10.1177/0022034519850812 31136721 PMC6616121

[B62] Shuhaibar N. Hand A. R. Terasaki M. (2021). Odontoblast processes of the mouse incisor are plates oriented in the direction of growth. Anat. Rec. 304, 1820–1827. 10.1002/ar.24570 PMC8359275 33190419

[B63] Strong A. L. Spreadborough P. J. Dey D. Yang P. Li S. Lee A. (2021). BMP ligand trap ALK3-fc attenuates osteogenesis and heterotopic ossification in blast-related lower extremity trauma. Stem Cells Dev. 30, 91–105. 10.1089/scd.2020.0162 33256557 PMC7826435

[B64] Szilágyi S. S. Amsalem-Zafran A. R. Shapira K. E. Ehrlich M. Henis Y. I. (2022). Competition between type I activin and BMP receptors for binding to ACVR2A regulates signaling to distinct Smad pathways. BMC Biol. 20, 50. 10.1186/s12915-022-01252-z 35177083 PMC8855587

[B65] Tan J. Zhang M. Hai Z. Wu C. Lin J. Kuang W. (2019). Sustained release of two bioactive factors from supramolecular hydrogel promotes periodontal bone regeneration. ACS Nano 13, 5616–5622. 10.1021/acsnano.9b00788 31059238

[B66] Tateiwa D. Nakagawa S. Tsukazaki H. Okada R. Kodama J. Kushioka J. (2021). A novel BMP-2-loaded hydroxyapatite/beta-tricalcium phosphate microsphere/hydrogel composite for bone regeneration. Sci. Rep. 11, 16924. 10.1038/s41598-021-96484-4 34413442 PMC8376985

[B67] Tazawa K. Ikeda H. Kawashima N. Okiji T. (2017). Transient receptor potential melastatin (TRPM) 8 is expressed in freshly isolated native human odontoblasts. Arch. Oral Biol. 75, 55–61. 10.1016/j.archoralbio.2016.12.007 28043013

[B68] Thrivikraman G. Athirasala A. Gordon R. Zhang L. Bergan R. Keene D. R. (2019). Rapid fabrication of vascularized and innervated cell-laden bone models with biomimetic intrafibrillar collagen mineralization. Nat. Commun. 10, 3520. 10.1038/s41467-019-11455-8 31388010 PMC6684598

[B69] Uribe P. Plakwicz P. Larsson L. Czochrowska E. Westerlund A. Ransjö M. (2018). Study on site-specific expression of bone formation and resorption factors in human dental follicles. Eur. J. Oral Sci. 126, 439–448. 10.1111/eos.12568 30216610 PMC6282833

[B70] van Houdt C. I. A. Koolen M. K. E. Lopez-Perez P. M. Ulrich D. J. O. Jansen J. A. Leeuwenburgh S. C. G. (2021). Regenerating critical size rat segmental bone defects with a self-healing hybrid nanocomposite hydrogel: Effect of bone condition and BMP-2 incorporation. Macromol. Biosci. 21, e2100088. 10.1002/mabi.202100088 34117838

[B71] Vukicevic S. Oppermann H. Verbanac D. Jankolija M. Popek I. Curak J. (2014). The clinical use of bone morphogenetic proteins revisited: A novel biocompatible carrier device OSTEOGROW for bone healing. Int. Orthop. 38, 635–647. 10.1007/s00264-013-2201-1 24352822 PMC3936094

[B72] Wang B. Mastrogiacomo S. Yang F. Shao J. Ong M. M. A. Chanchareonsook N. (2019). Application of BMP-bone cement and FGF-gel on periodontal tissue regeneration in nonhuman primates. Tissue Eng. Part C Methods 25, 748–756. 10.1089/ten.TEC.2019.0160 31701811 PMC6998056

[B73] Wang J. Muir A. M. Ren Y. Massoudi D. Greenspan D. S. Feng J. Q. (2017). Essential roles of bone morphogenetic protein-1 and mammalian tolloid-like 1 in postnatal root dentin formation. J. Endod. 43, 109–115. 10.1016/j.joen.2016.09.007 27847137 PMC5164841

[B74] Wang K. Xu C. Xie X. Jing Y. Chen P. J. Yadav S. (2022). Axin2+ PDL cells directly contribute to new alveolar bone formation in response to orthodontic tension force. J. Dent. Res. 101, 695–703. 10.1177/00220345211062585 35001706 PMC9124907

[B75] Wang M. Fan J. Wang A. Jin X. Zhang Z. Hu X. (2023). Effect of local application of bone morphogenetic protein -2 on experimental tooth movement and biological remodeling in rats. Front. Physiol. 14, 1111857. 10.3389/fphys.2023.1111857 37143931 PMC10151543

[B76] Wang S. Li J. Sun H. Sha L. Guo Y. Gu G. (2020). Treatment with soluble bone morphogenetic protein type 1A receptor fusion protein alleviates irradiation-induced bone loss in mice through increased bone formation and reduced bone resorption. Am. J. Transl. Res. 12, 743–757. 32269709 PMC7137047

[B77] Wang T. Zhang X. Bikle D. D. (2017). Osteogenic differentiation of periosteal cells during fracture healing. J. Cell. Physiol. 232, 913–921. 10.1002/jcp.25641 27731505 PMC5247290

[B78] Wang W. Dang M. Zhang Z. Hu J. Eyster T. W. Ni L. (2016). Dentin regeneration by stem cells of apical papilla on injectable nanofibrous microspheres and stimulated by controlled BMP-2 release. Acta Biomater. 36, 63–72. 10.1016/j.actbio.2016.03.015 26971664 PMC4846535

[B79] Wang Y. Cox M. K. Coricor G. MacDougall M. Serra R. (2013). Inactivation of Tgfbr2 in Osterix-Cre expressing dental mesenchyme disrupts molar root formation. Dev. Biol. 382, 27–37. 10.1016/j.ydbio.2013.08.003 23933490 PMC3783640

[B80] Wen W. Que K. Zang C. Wen J. Sun G. Zhao Z. (2017). Expression and distribution of three transient receptor potential vanilloid(TRPV) channel proteins in human odontoblast-like cells. J. Mol. Histol. 48, 367–377. 10.1007/s10735-017-9735-2 28905239

[B81] Wu M. Chen G. Li Y. P. (2016). TGF-β and BMP signaling in osteoblast, skeletal development, and bone formation, homeostasis and disease. Bone Res. 4, 16009. 10.1038/boneres.2016.9 27563484 PMC4985055

[B82] Xin Y. Zhao N. Wang Y. (2022). Multiple roles of runt-related transcription factor-2 in tooth eruption: bone formation and resorption. Arch. Oral Biol. 141, 105484. 10.1016/j.archoralbio.2022.105484 35749976

[B83] Yan Z. Q. Wang X. K. Zhou Y. Wang Z. G. Wang Z. X. Jin L. (2020). H-type blood vessels participate in alveolar bone remodeling during murine tooth extraction healing. Oral Dis. 26, 998–1009. 10.1111/odi.13321 32144839

[B84] Yang W. Harris M. A. Cui Y. Mishina Y. Harris S. E. Gluhak-Heinrich J. (2012). Bmp2 is required for odontoblast differentiation and pulp vasculogenesis. J. Dent. Res. 91, 58–64. 10.1177/0022034511424409 21984706 PMC3232115

[B85] Yuan G. Yang G. Zheng Y. Zhu X. Chen Z. Zhang Z. (2015). The non-canonical BMP and Wnt/β-catenin signaling pathways orchestrate early tooth development. Development 142, 128–139. 10.1242/dev.117887 25428587 PMC4299140

[B86] Yumoto H. Hirao K. Hosokawa Y. Kuramoto H. Takegawa D. Nakanishi T. (2018). The roles of odontoblasts in dental pulp innate immunity. Jpn. Dent. Sci. Rev. 54, 105–117. 10.1016/j.jdsr.2018.03.001 30128058 PMC6094490

[B87] Zang S. Mu R. Chen F. Wei X. Zhu L. Han B. (2019). Injectable chitosan/β-glycerophosphate hydrogels with sustained release of BMP-7 and ornidazole in periodontal wound healing of class III furcation defects. Mat. Sci. Eng. C 99, 919–928. 10.1016/j.msec.2019.02.024 30889766

[B88] Zeng L. He H. Sun M. Gong X. Zhou M. Hong Y. (2022). Runx2 and Nell-1 in dental follicle progenitor cells regulate bone remodeling and tooth eruption. Stem Cell Res. Ther. 13, 486. 10.1186/s13287-022-03140-3 36175952 PMC9524038

[B89] Zhang H. Zhang Y. Terajima M. Romanowicz G. Liu Y. Omi M. (2020). Loss of BMP signaling mediated by BMPR1A in osteoblasts leads to differential bone phenotypes in mice depending on anatomical location of the bones. Bone 137, 115402. 10.1016/j.bone.2020.115402 32360900 PMC7354232

[B90] Zhang Y. McNerny E. G. Terajima M. Raghavan M. Romanowicz G. Zhang Z. (2016). Loss of BMP signaling through BMPR1A in osteoblasts leads to greater collagen cross-link maturation and material-level mechanical properties in mouse femoral trabecular compartments. Bone 88, 74–84. 10.1016/j.bone.2016.04.022 27113526 PMC4899267

[B91] Zhu L. Ma J. Mu R. Zhu R. Q. Chen F. Wei X. C. (2018). Bone morphogenetic protein 7 promotes odontogenic differentiation of dental pulp stem cells *in vitro* . Life Sci. 202, 175–181. 10.1016/j.lfs.2018.03.026 29555587

